# Prediction of death and prolonged mechanical ventilation in acute lung injury

**DOI:** 10.1186/cc5909

**Published:** 2007-05-10

**Authors:** Ognjen Gajic, Bekele Afessa, B Taylor Thompson, Fernando Frutos-Vivar, Michael Malinchoc, Gordon D Rubenfeld, André Esteban, Antonio Anzueto, Rolf D Hubmayr, the Second International Study of Mechanical Ventilation and ARDS-net Investigators

**Affiliations:** 1 Mayo Clinic, 200 First Street SW, Rochester, Minnesota, 55905, USA; 2 Massachusetts General Hospital, 55 Fruit Street, Boston, Massachusetts, 02114, USA; 3 Hospital Universitario de Getafe, Carretera de Toledo km 12,500, 28905 Getafe, Madrid, Spain; 4 University of Washington, Harborview Medical Center, 325 Ninth Avenue, Campus Box 359762, Seattle, Washington, 98104, USA; 5 University of Texas Health Science Center,7703 Floyd Curl Drive, San Antonio, Texas, 78229, USA

## Abstract

**Introduction:**

Prediction of death and prolonged mechanical ventilation is important in terms of projecting resource utilization and in establishing protocols for clinical studies of acute lung injury (ALI). We aimed to identify risk factors for a combined end-point of death and/or prolonged ventilator dependence and developed an ALI-specific prediction model.

**Methods:**

In this retrospective analysis of three multicenter clinical studies, we identified predictors of death or ventilator dependence from variables prospectively recorded during the first three days of mechanical ventilation. After the prediction model was derived in an international cohort of patients with ALI, it was validated in two independent samples of patients enrolled in a clinical trial involving 17 academic centers and a North American population-based cohort.

**Results:**

A combined end-point of death and/or ventilator dependence at 14 days or later occurred in 68% of patients in the international cohort, 60% of patients in the clinical trial, and 59% of patients in the population-based cohort. In the derivation cohort, a model based on age, oxygenation index on day 3, and cardiovascular failure on day 3 predicted death and/or ventilator dependence. The prediction model performed better in the clinical trial validation cohort (area under the receiver operating curve 0.81, 95% confidence interval 0.77 to 0.84) than in the population-based validation cohort (0.71, 95% confidence interval 0.65 to 0.76).

**Conclusion:**

A model based on age and cardiopulmonary function three days after the intubation is able to predict, moderately well, a combined end-point of death and/or prolonged mechanical ventilation in patients with ALI.

## Introduction

Although a significant number of patients with acute lung injury (ALI) die or require prolonged mechanical ventilation, the tools for predicting mortality and morbidity in this group of patients are limited [1,2]. Parameters related to the degree of impairment in pulmonary function and nonpulmonary organ failures have been associated with increased mortality and prolonged mechanical ventilation in patients with ALI, and in mechanically ventilated patients in general [1-11]. Compared with values collected on day 1, evolution of the disease and response to treatment during the first three days of mechanical ventilation provide valuable prognostic information [1,2,12].

The present study analyzed potential predictors of outcome from mechanical ventilation in patients with ALI in three recent prospective cohorts with the following specific aims: to identify risk factors for death and/or ventilator dependence; to develop an ALI-specific prediction model; and to validate the prediction model in independent samples from both population-based and clinical trial databases, in order to determine the potential value of the model for clinical decision making and clinical trial design in patients who are likely to die or require prolonged mechanical ventilation.

## Materials and methods

In this retrospective study, we used data from patients with ALI enrolled in three recent prospective cohorts. The detailed protocols of these three studies, namely the Second International Study of Mechanical Ventilation (VENTILA) [13], the ARDS-net clinical trial (low tidal volume [14] and lisophylline [15]), and the King County Lung Injury Project (KCLIP) [16], have previously been reported. The studies were approved by local ethics committees in each participating institution. ALI and acute respiratory distress syndrome (ARDS) were defined according to the American-European Consensus conference [17] in all three cohorts.

### Outcome measures

The main outcome of interest was the composite outcome of death in hospital and/or ventilator dependence for more than two weeks after intubation (less than 14 ventilator-free days). There are a number of reasons why we selected the combined end-point of death and/or ventilator dependence. First, specific intensive care unit interventions that may be applied at the bedside or tested in a clinical trial may affect both survival and the duration of mechanical ventilation. Second, during the first few days of mechanical ventilation it may be difficult to discriminate between patients who will die in the hospital and those who require prolonged mechanical ventilation but ultimately will survive hospitalization. Third, the fact that a significant proportion of survivors of prolonged mechanical ventilation experience a long-term decrease in quality of life may be particularly important in the informed consent process and end-of life discussions in patients with respiratory failure who are at high risk for death or prolonged mechanical ventilation. Finally, analogous to the concept of ventilator-free days, the combined end-point may be a more sensitive outcome for the design of clinical trials testing specific therapeutic interventions.

### Patient groups

#### Derivation cohort

From the VENTILA study database, we identified patients with ALI who were alive and mechanically ventilated through an endotracheal tube for at least three days. Patients who died, who underwent earlier tracheostomy, or who were noninvasively ventilated on or before day 3 after initial intubation were excluded.

#### Validation cohorts

Patients with ALI enrolled into the two ARDS-net studies (clinical trial sample) and KCLIP study (population-based sample), who were alive and mechanically ventilated through an endotracheal tube on day 3 after intubation, were identified. Those who died or were ventilated noninvasively during the first three days after initial intubation were excluded. Tracheostomy data were not available in the validation cohorts.

### Measures and parameters recorded

A number of variables, prospectively collected during the first three days of mechanical ventilation, were abstracted from the databases. Baseline characteristics abstracted included age, sex, body mass index, severity of illness (Simplified Acute Physiology Score [SAPS] II [18] and Sequential Organ Failure Assessment [19]), and underlying ALI risk factors (pulmonary and extrapulmonary). Respiratory variables included peak and plateau airway pressures, positive end-expiratory pressure (PEEP), arterial oxygen tension (PaO_2_)/fractional inspired oxygen (FiO_2_) ratio, arterial carbon dioxide tension (PaCO_2_), oxygenation index [8], and minute volume needed to bring PaCO_2 _to 40 mmHg (VE40) [6]. The following measures of nonpulmonary organ failures were also abstracted: serum creatinine (kidney), serum bilirubin (liver), platelet count (hematologic variable), Glasgow Coma Scale score (neurologic variable), and (arterial hypotension or the use of vasopressors (cardiovascular variable).

Oxygenation index was calculated using the following formula: mean airway pressure × FiO_2_/PaO_2_. Mean airway pressure was calculated as (peak airway pressure + PEEP)/2. VE40 was calculated as (minute volume × PaCO_2_)/40. Cardiovascular failure was defined as systolic blood pressure less than 90 mmHg or the use of vasopressors, defined as follows: > 5 μg/kg per min dopamine or any dose of norepinephrine (noradrenaline), epinephrine (adrenalilne), vasopressin, or phenylephrine.

### Statistical analysis

Data are summarized as median (interquartile range) or as proportions. Univariate logistic regression analysis and recursive partitioning were used to identify variables associated with increased risk for death or ventilator dependence in the derivation cohort. Stepwise multiple logistic regression identified combination of variables with the best predictive ability. Variables were included in the model if they were biologically plausible and associated with the outcome of interest in univariate analysis (*P *< 0.1 or odds ratio ≥ 2.0 for nominal variables, or a median split of continuous variables). The final model was selected by backward elimination of nonsignificant variables. Hosmer-Lemeshow statistics [20] were used to determine the calibration of the model in each sample. Receiver operating characteristic curves were plotted and area under the curve for the prediction model was compared with those of general severity scores measured in each of the cohorts. Two cutoff scores (one more sensitive for clinical trial design and one more specific for clinical practice and estimating resource utilization) were identified in the derivation cohort and were subsequently validated, with calculation of positive and negative likelihood ratios for both cut-off scores. Where appropriate, odds ratios (ORs) and 95% confidence intervals (CIs) were calculated. *P *< 0.05 was considered statistically significant. SAS statistical software (SAS Institute, Cary, NS, USA) was used in all data analyses.

## Results

**Figure 1:**
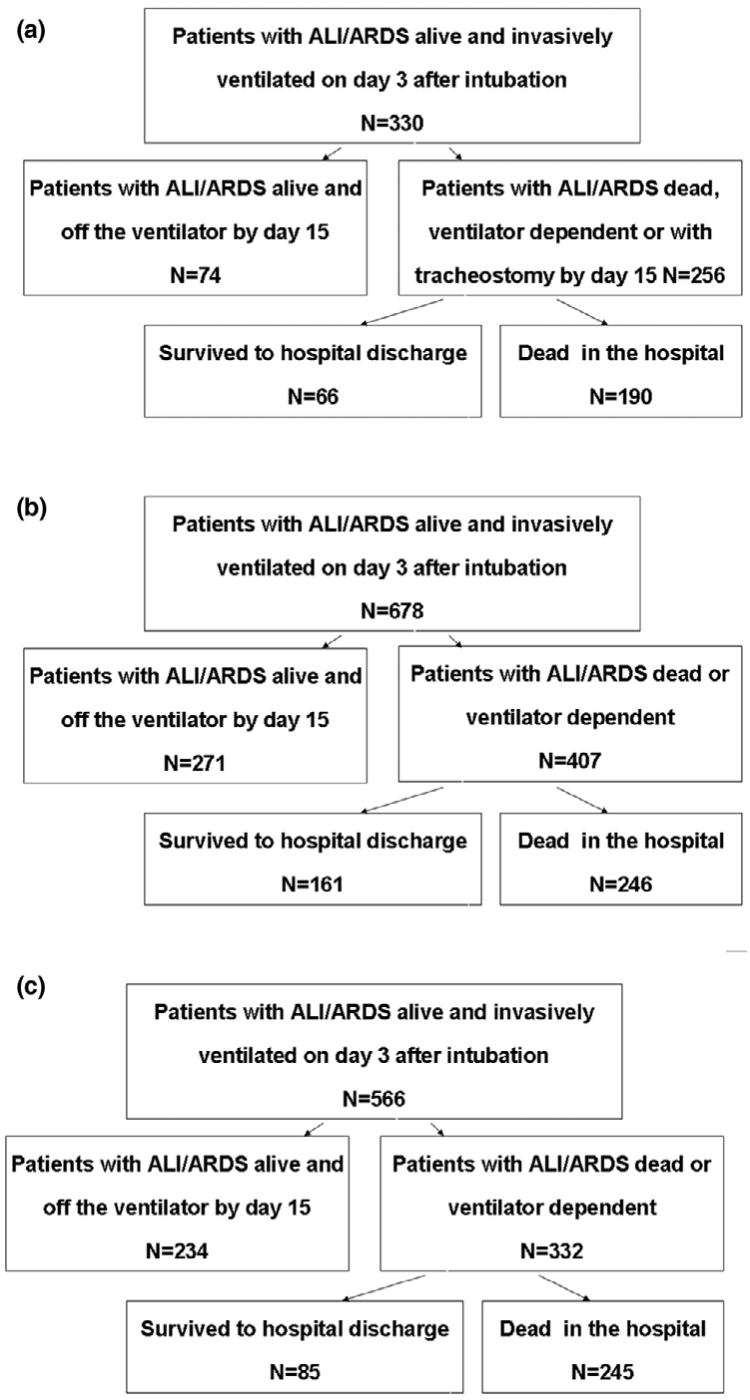
Outline of the study. Shown are **(a) **the derivation cohort, **(b) **validation cohort (clinical trial), **(c) **validation cohort (population based). ALI, acute lung injury; ARDS, acute respiratory distress syndrome.

The primary outcome (death and/or ventilator dependence for longer than 14 days) occurred in 68% of patients in the international derivation cohort (VENTILA), in 60% of patients in the clinical trial validation cohort (ARDS-net), and in 59% of patients in the population-based validation cohort (KCLIP; Figure 1). Hospital mortality was 58% in VENTILA, 36% in ARDS-net, and 43% in KCLIP.

**Figure 2:**
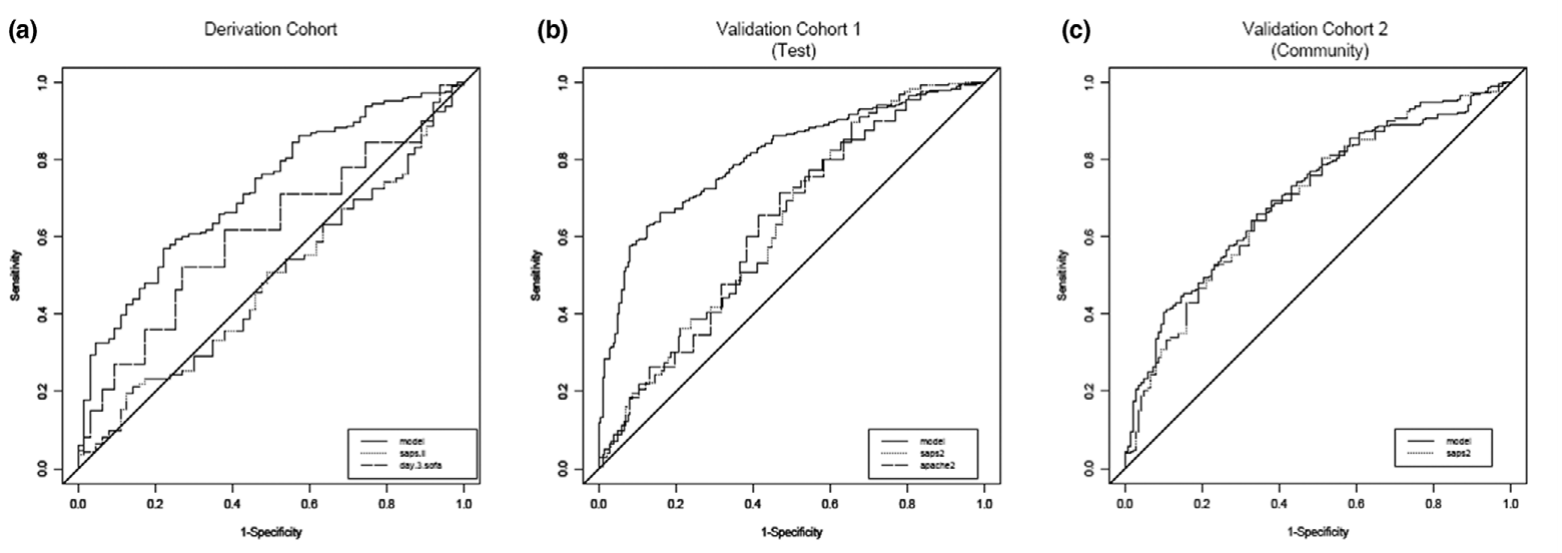
Area under receiving operator curves: model versus day 1 SAPS II and day 3 SOFA scores. **(a) **International derivation cohort (VENTILA), **(b) **clinical trial validation cohort (ARDS-Net), and **(c) **population-based validation cohort (KCLIP). SAPS, Simplified Acute Physiology Score; SOFA, Sequential Organ Failure Assessment.

Table 1 shows the association of the predictor variables with death and/or ventilator dependence using univariate analyses in the derivation cohort. A simple logistic regression model (0.03 × age + 0.07 × day 3 oxygenation index + day 3 cardiovascular failure [1 if present, 0 if absent]) had moderate discriminative power and was well calibrated (Table 2, Figure 2 and Additional file 1).

**Table 1:** Baseline and day 3 characteristics of patients in the derivation cohort

Characteristic	Alive and not ventilator dependent by day 15	Dead, ventilator-dependent, or with tracheostomy by day 15	*P*
Age	53 (40 to 66)	63 (49 to 72)	0.004
Female sex (*n *[%])	34 (46)	87 (34)	0.063
BMI (kg/m^2^)	27 (24 to 31)	26 (23 to 31)	0.138
SAPS II score	46 (37 to 58)	47 (35 to 59)	0.760
Sepsis (*n *[%])	28 (38%)	99 (39%)	0.897
Extrapulmonary cause of ALI/ARDS (*n *[%])	29 (27%)	45 (20%)	0.205
Day 3 PaCO_2 _(mmHg)	39 (33 to 44)	41 (36 to 47)	0.012
Day 3 PaO_2_/FiO_2_	174 (146 to 195)	153 (105 to 198)	0.013
Day 3 Vt (ml/kg PBW)	9.1 (7.2 to 10.6)	8.5 (7.1 to 10.4)	0.181
Day 3 Ppl (cmH_2_O)	23 (19 to 26)	26 (21 to 30)	0.012
Day 3 Ppk (cmH_2_O)	28 (26 to 32)	31 (27 to 38)	0.010
Day 3 PEEP (cmH_2_O)	5 (5 to 9)	8 (5 to 12)	0.003
Day 3 VE40 (ml/kg PBW/min)	142 (109 to 186)	162 (128 to 214)	0.006
Day 3 oxygenation index	10.4 (8.2 to 14.7)	13.9 (9.3 to 23.1)	0.001
Day 3 ΔPEEP (cmH_2_O)^a^	0 (-2 to 0)	0 (0 to 3)	< 0.001
Day 3 SOFA score	9 (7 to 10)	10 (7 to 13)	0.055
Day 3 shock (*n *[%])	14 (19)	99 (39)	0.001

The values are presented as median (interquartile range) for continuous variables and number (%) for categorical variables. ALI, acute lung injury/acute respiratory distress syndrome; BMI, body mass index; FiO_2_, fractional inspired oxygen; PaCO_2_, arterial carbon dioxide tension; PaO_2_, arterial oxygen tension; PBW, predicted body weight; PEEP, positive end-expiratory pressure; Ppk, peak airway pressure; Ppl, plateau airway pressure; SAPS, Simplified Acute Physiology Score; SOFA, Sequential Organ Failure Assessment, VE40, minute ventilation required to achieve PaCO_2 _of 40 mmHg; Vt, tidal volume. ^a^PEEP at day 3 minus PEEP at day 1.

**Table 2:** Performance of the prediction model

Study cohort	Discrimination^a^	Calibration^b^
Derivation		
VENTILA	0.72 (0.65 to 0.79)	0.78
Validation		
ARDS-Net	0.81 (0.77–0.84)	0.12
KCLIP	0.71 (0.65–0.76)	0.03

^a^Area under the receiver operating curve (95% confidence interval). ^b^Hosmer-Lemeshow goodness of fit (*P *value).

In the clinical trial validation cohort, the model predicted death or ventilator dependence better than day 1 SAPS II and Acute Physiology and Chronic Health Evaluation (APACHE) II scores (*P *< 0.01; Figure 2). The discriminative power and calibration were good (Table 2). In the population-based validation cohort, the model was less well calibrated and performed similar to day 1 SAPS II score (Table 2 and Figure 2).

Both more sensitive (>3.0) and more specific (>3.5) cutoff scores for the model were identified in the derivation cohort and subsequently validated in the two validation cohorts (Table 3). Positive and negative likelihood ratios for different cutoff points of the model and for day 3 values of oxygenation index and PaO_2_/FiO_2 _ratio are presented in Table 3. Missing data precluded calculation of oxygenation index in 16% of patients in the derivation (VENTILA) cohort, 25% in the ARDS-net cohort, and 35% in the KCLIP cohort, and these patients were excluded from the analysis.

**Table 3:** Positive and negative likelihood ratios for predicting death or more than 14 days of ventilator dependency

Patient population	Diagnostic modality	Statistic	Estimate (95% CI)
VENTILA	Model (≤ 3.0)	LR+	2.52 (1.57 to 4.07)
		LR-	0.56 (0.46 to 0.69)
	Model (≤ 3.5)	LR+	3.53 (1.72 to 7.24)
		LR-	0.68 (0.59 to 0.79)
	Oxygenation index (≤ 15)	LR+	1.98 (1.22 to 3.21)
		LR-	0.72 (0.6 to 0.86)
	PaO_2_/FiO_2 _(≤ 150)	LR+	1.77 (1.2 to 2.62)
		LR-	0.70 (0.58 to 0.85)
ARDS-net	Model (≤ 3)	LR+	3.15 (2.38 to 4.19)
		LR-	0.40 (0.33 to 0.48)
	Model (≤ 3.5)	LR+	6.86 (4.1 to 11.47)
		LR-	0.51 (0.46 to 0.58)
	Oxygenation index (≤ 15)	LR+	2.18 (1.71 to 2.77)
		LR-	0.52 (0.44 to 0.62)
	PaO_2_/FiO_2 _(≤ 150)	LR+	1.98 (1.6 to 2.45)
		LR-	0.57 (0.49 to 0.66)
KCLIP	Model (≤ 3)	LR+	1.56 (1.3 to 1.87)
		LR-	0.45 (0.34 to 0.6)
	Model (≤ 3.5)	LR+	1.83 (1.42 to 2.37)
		LR-	0.58 (0.47 to 0.71)
	Oxygenation index (≤ 15)	LR+	1.75 (1.33 to 2.32)
		LR-	0.67 (0.56 to 0.8)
	PaO_2_/FiO_2 _(≤ 150)	LR+	2.22 (1.69 to 2.92)
		LR-	0.62 (0.54 to 0.72)

CI, confidence interval; FiO_2_, fractional inspired oxygen; LR+, positive likelihood ratios; LR-, negative likelihood ratios; PaO_2_, arterial oxygen tension.

## Discussion

In this retrospective study of three recent, large cohorts of patients with ALI, we observed that two-thirds of patients who were alive and invasively ventilated on day 3 after endotracheal intubation reached the composite outcome of death and/or ventilator dependence for more than two weeks. A simple model derived from age and cardiopulmonary function three days after intubation predicted death and/or ventilator dependence quite well in patients who were cared for in academic centers and enrolled in one of the ARDS-net trials. The model performance was acceptable, but not as strong when applied to the US population based cohort.

Altered lung mechanics and abnormal gas exchange are hallmarks of impaired lung function in ALI and are of prognostic significance [3]. Most models for quantifying gas exchange in a clinical setting consider the lungs as having three compartments: a shunt compartment, a dead space compartment, and normal lung. The size of the shunt compartment is commonly estimated by the PaO_2_/FiO_2 _ratio, whereas that of the dead space compartment scales with dead space ventilation (Vd/Vt) [3] and VE40 [6]. Both parameters are exquisitely sensitive to cardiac output and ventilator management. To adjust for the latter and to account for abnormal respiratory mechanics, clinicians at times derive an oxygenation index, which is defined as the PaO_2_/FiO_2 _ratio normalized by mean airway pressure. Oxygenation index has been associated with outcome in both adults and children with ALI [7,8]. Apart from oxygenation index, other parameters relating to the ventilator (PEEP and plateau pressure) or gas exchange (PaCO_2 _and VE40) did not significantly contribute to the discriminative power of our model.

The presence of persistent shock, renal failure, age, immunosuppression, underlying cause of ALI, and overall severity of illness were previously identified as important nonpulmonary outcome determinants [1,2,4,5,10,21,22]. In the ARDS-net low tidal volume study [14], age, APACHE II score, plateau pressure, the number of organ failures (using the Brussels Organ Failure Classification), number of hospital days before enrollment, and arterial-alveolar oxygen gradient were found to be independent prognostic factors, and were used in the mortality adjustments reported in the recent ARDS-net study [11]. Age by itself is known to be an important predictor of poor outcome in patients with ALI [23]. Except for age and day 3 cardiovascular failure, additional markers of nonpulmonary organ failures (creatinine, platelet count, bilirubin, and Glasgow Coma Scale score) did not contribute to the discriminative power of our model. A logistic model similar to ours and based on age and day 3 oxygenation impairment was found to be predictive of prolonged mechanical ventilation in burn patients [24].

Of note, our model had better discrimination in a clinical trial dataset than in the two observational cohorts. This suggests that unmeasured factors related to co-interventions such as ventilator management or weaning, end of life care, and co-morbidities may introduce heterogeneity in patients who meet ALI definition in observational studies. Nevertheless, the model discrimination did not worsen when it was evaluated in the real-world setting of a population-based cohort of patients.

One of the objectives of our prediction model was to aid in decision making and clinical trial design with regard to the timing of tracheostomy. In a recent clinical trial conducted by Rumbak and coworkers [25], patients randomly assigned to early tracheostomy not only had shorter duration of mechanical ventilation and intensive care unit length of stay but also markedly lower hospital mortality (31.7% versus 61.7%; *P *< 0.005). Although the authors did not specifically address how many of these patients met criteria for ALI, it is likely, based on the description of the patient population, that a significant number of patients did indeed have ALI. One of the main criticisms of this study included somewhat arbitrary prediction of the need for prolonged mechanical ventilation (APACHE II score > 25). We believe that our model could be used in future studies of early versus late tracheostomy in patients with ALI.

The principal limitations or our study stem from its retrospective design insofar as neither of the original studies were designed to answer our questions. It is possible that some variables that were not routinely collected, for example Vd/Vt or net fluid balance, might have added to the model. Missing data precluded calculation of the oxygen index and, therefore, of the model for a significant number of patients. Having missing data did not significantly influence the outcome in the derivation cohort (OR 1.12, 95% CI 0.56 to 2.41). In the validation cohorts, missing data were associated with a lower risk for death or prolonged ventilation (OR 0.55, 95% CI 0.39 to 0.78 in the ARDS-net sample; OR 0.64, 95% CI 0.45 to 0.90 in the KCLIP sample). Although the design of our study does not allow us to state the reasons for the missing data, we speculate that patients in whom the data needed to calculate oxygenation index were lacking (mean airway pressure and FiO_2_/PaO_2_) may have been improving clinically and undergoing weaning attempts.

Although our choice of a combined outcome including death and prolonged mechanical ventilation as the primary outcome may be questioned, it is important to emphasize that the two may not be reliably differentiated during the first few days of mechanical ventilation. The distinction between patients who die and those who undergo prolonged mechanical ventilation could be related to the preferences of patients and physicians regarding withholding of prolonged ventilation and rehabilitation, bearing in mind the potential poor quality of life in the future that may result from such interventions. Among the patients who survive the first few days of mechanical ventilation, the mortality and prolonged mechanical ventilation may be viewed as different ends of the spectrum of poor prognosis in patients with ALI. Improvement in the accuracy of prediction in future prospective studies will require careful consideration not only of factors related to underling pulmonary and nonpulmonary organ dysfunction but also of the characteristics of individual practices, patient preferences, premorbid functional status, and, possibly, biomarkers of lung injury and systemic inflammation.

## Conclusion

A majority of patients with ALI are at risk for death or prolonged mechanical ventilation. A model derived from age, oxygenation index, and cardiovascular failure three days after intubation predicts death or prolonged mechanical ventilation and may inform decisions regarding specific interventions such as tracheostomy, particularly in terms of clinical trial design. However, because of the retrospective design of the present study, a validation study is warranted in an independent sample of patients.

## Key messages

• A substantial number of patients with ALI reached the combined end-point of death in the hospital or prolonged mechanical ventilation.

• A simple model consisting of age and cardiopulmonary function on day 3 of mechanical ventilation predicted death and/or prolonged mechanical ventilation in patients with ALI.

• Performance of the prediction model was better in the population of patients enrolled in a clinical trial than in the community.

## Abbreviations

ALI = acute lung injury; APACHE = Acute Physiology and Chronic Health Evaluation; ARDS = acute respiratory distress syndrome; CI = confidence interval; FiO_2 _= fractional inspired oxygen; OR = odds ratio; PaCO_2 _= arterial carbon dioxide tension; PaO_2 _= arterial oxygen tension; PEEP = positive end-expiratory pressure; SAPS = Simplified Acute Physiology Score; VE40 = minute volume needed to bring PaCO_2 _to 40 mmHg.

## Competing interests

The authors declare that they have no competing interests.

## Authors' contributions

OG, BA, RDH, GDR, and FF-V contributed to the study design, and data analysis and interpretation. OG, BTT, GDR, FF-V, AA, and AE contributed to the data collection and interpretation. MM contributed to data analysis and interpretation.

## Supplementary Material

Additional file 1 A Word document providing additional statistical details and summarizing the investigators who participated in VENTILA (Second International Study of Mechanical Ventilation), by country and the National Heart, Lung, and Blood Institute (NHLBI) ARDS Clinical Trials Network. Click here for file
