# Supplementary material for: Prediction of death and prolonged mechanical ventilation in acute lung injury
Source: Crit Care. 2007 May 10;11(3):R53. doi: 10.1186/cc5909 (PMC2206401; doi:10.1186/cc5909)
Supplement: Additional file 1 — A Word document providing additional statistical details and summarizing the investigators who participated in VENTILA (Second International Study of Mechanical Ventilation), by country and the National Heart, Lung, and Blood Institute (NHLBI) ARDS Clinical Trials Network. [file cc5909-S1.doc]

**Prediction of death and prolonged mechanical ventilation in acute lung injury**

**Online data supplement**

Table A: Statistical software output demonstrating the calibration of the model in 1) derivation cohort (VENTILA), 2) clinical trial validation cohort (ARDS-net) and 3) community validation cohort (KCLIP)

1)

| **Model Information** | | |
| --- | --- | --- |
| **Data Set** | VENTILA |  |
| **Response Variable** | Composite outcome |  |
| **Number of Response Levels** | 2 |  |
| **Model** | binary logit |  |
| **Optimization Technique** | Fisher's scoring |  |

| **Association of Predicted Probabilities and Observed Responses** | | | |
| --- | --- | --- | --- |
| **Percent Concordant** | 71.6 | **Somers' D** | 0.435 |
| **Percent Discordant** | 28.1 | **Gamma** | 0.437 |
| **Percent Tied** | 0.3 | **Tau-a** | 0.153 |
| **Pairs** | 13482 | **c** | 0.718 |

| **Partition for the Hosmer and Lemeshow Test** | | | | | |
| --- | --- | --- | --- | --- | --- |
| **Group** | **Total** | **Composite outcome = 1** | | **Composite outcome = 2** | |
|  |  | **Observed** | **Expected** | **Observed** | **Expected** |
| **1** | 28 | 13 | 14.54 | 15 | 13.46 |
| **2** | 28 | 16 | 17.58 | 12 | 10.42 |
| **3** | 28 | 22 | 19.20 | 6 | 8.80 |
| **4** | 28 | 22 | 20.26 | 6 | 7.74 |
| **5** | 28 | 19 | 21.36 | 9 | 6.64 |
| **6** | 28 | 23 | 22.40 | 5 | 5.60 |
| **7** | 28 | 24 | 23.71 | 4 | 4.29 |
| **8** | 28 | 24 | 24.86 | 4 | 3.14 |
| **9** | 28 | 27 | 25.85 | 1 | 2.15 |
| **10** | 25 | 24 | 24.24 | 1 | 0.76 |

| **Hosmer and Lemeshow Goodness-of-Fit Test** | | |
| --- | --- | --- |
| **Chi-Square** | **DF** | **Pr > ChiSq** |
| 4.7774 | 8 | 0.7811 |

| **Model Information** | | |
| --- | --- | --- |
| **Data Set** | ARDS-net |  |
| **Response Variable** | Composite outcome |  |
| **Number of Response Levels** | 2 |  |
| **Model** | binary logit |  |
| **Optimization Technique** | Fisher's scoring |  |

| **Association of Predicted Probabilities and Observed Responses** | | | |
| --- | --- | --- | --- |
| **Percent Concordant** | 80.5 | **Somers' D** | 0.612 |
| **Percent Discordant** | 19.3 | **Gamma** | 0.613 |
| **Percent Tied** | 0.2 | **Tau-a** | 0.283 |
| **Pairs** | 58926 | **c** | 0.806 |

| **Partition for the Hosmer and Lemeshow Test** | | | | | |
| --- | --- | --- | --- | --- | --- |
| **Group** | **Total** | **Composite outcome = 1** | | **Composite outcome = 2** | |
|  |  | **Observed** | **Expected** | **Observed** | **Expected** |
| **1** | 51 | 14 | 10.78 | 37 | 40.22 |
| **2** | 51 | 18 | 17.82 | 33 | 33.18 |
| **3** | 51 | 19 | 22.49 | 32 | 28.51 |
| **4** | 51 | 29 | 26.66 | 22 | 24.34 |
| **5** | 51 | 28 | 31.23 | 23 | 19.77 |
| **6** | 51 | 30 | 35.87 | 21 | 15.13 |
| **7** | 51 | 46 | 40.48 | 5 | 10.52 |
| **8** | 51 | 46 | 44.21 | 5 | 6.79 |
| **9** | 51 | 48 | 47.26 | 3 | 3.74 |
| **10** | 46 | 44 | 45.20 | 2 | 0.80 |

| **Hosmer and Lemeshow Goodness-of-Fit Test** | | |
| --- | --- | --- |
| **Chi-Square** | **DF** | **Pr > ChiSq** |
| 12.8954 | 8 | 0.1155 |

| **Model Information** | | |
| --- | --- | --- |
| **Data Set** | KCLIP |  |
| **Response Variable** | Composite_Outcome |  |
| **Number of Response Levels** | 2 |  |
| **Model** | binary logit |  |
| **Optimization Technique** | Fisher's scoring |  |

| **Association of Predicted Probabilities and Observed Responses** | | | |
| --- | --- | --- | --- |
| **Percent Concordant** | 70.5 | **Somers' D** | 0.414 |
| **Percent Discordant** | 29.1 | **Gamma** | 0.415 |
| **Percent Tied** | 0.3 | **Tau-a** | 0.194 |
| **Pairs** | 31373 | **c** | 0.707 |

| **Partition for the Hosmer and Lemeshow Test** | | | | | |
| --- | --- | --- | --- | --- | --- |
| **Group** | **Total** | **Composite Outcome = 1** | | **Composite Outcome = 2** | |
|  |  | **Observed** | **Expected** | **Observed** | **Expected** |
| **1** | 37 | 18 | 12.92 | 19 | 24.08 |
| **2** | 37 | 9 | 16.51 | 28 | 20.49 |
| **3** | 37 | 20 | 18.89 | 17 | 18.11 |
| **4** | 37 | 20 | 20.54 | 17 | 16.46 |
| **5** | 37 | 24 | 22.37 | 13 | 14.63 |
| **6** | 37 | 24 | 24.23 | 13 | 12.77 |
| **7** | 37 | 21 | 25.61 | 16 | 11.39 |
| **8** | 37 | 33 | 27.28 | 4 | 9.72 |
| **9** | 37 | 30 | 30.10 | 7 | 6.90 |
| **10** | 33 | 30 | 30.54 | 3 | 2.46 |

| **Hosmer and Lemeshow Goodness-of-Fit Test** | | |
| --- | --- | --- |
| **Chi-Square** | **DF** | **Pr > ChiSq** |
| 17.1095 | 8 | 0.0290 |

Table B Statistical software output demonstrating the area under the receiver operator curve for the model and the comparisons with SAPS2 and day 3 SOFA in VENTILA cohort, SAPS2 and APACHE II in ARDS-net cohort and SAPS2 in KCLIP cohort

**One sided tests for AUC > 0.5**

| VENTILA | | | | | | |
| --- | --- | --- | --- | --- | --- | --- |
|  | ROC AUC | Std. Error | z value | Pr(>z) | lower.95 | upper.95 |
| model | 0.7178 | 0.0348 | 6.25 | 0.0000 | 0.6495 | 0.7861 |
| saps2 | 0.5270 | 0.0402 | 0.67 | 0.2511 | 0.4482 | 0.6057 |
| day.3.sofa | 0.5716 | 0.0384 | 1.86 | 0.0311 | 0.4964 | 0.6469 |

| ARDS-net | | | | | | |
| --- | --- | --- | --- | --- | --- | --- |
|  | ROC AUC | Std. Error | z value | Pr(>z) | lower.95 | upper.95 |
| model | 0.8058 | 0.0194 | 15.80 | 0 | 0.7679 | 0.8437 |
| saps2 | 0.6254 | 0.0267 | 4.69 | 0 | 0.5730 | 0.6778 |
| apache2 | 0.6103 | 0.0267 | 4.13 | 0 | 0.5580 | 0.6627 |

| KCLIP | | | | | | |
| --- | --- | --- | --- | --- | --- | --- |
|  | ROC AUC | Std. Error | z value | Pr(>z) | lower.95 | upper.95 |
| model | 0.7068 | 0.0274 | 7.55 | 0 | 0.6531 | 0.7604 |
| saps2 | 0.6906 | 0.0284 | 6.72 | 0 | 0.6350 | 0.7462 |

**Pair-wise comparisons**

| VENTILA | | | | |
| --- | --- | --- | --- | --- |
|  | Area Diff | P-value | lower.95 | upper.95 |
| saps2 - model | -0.1909 | 0.0002 | -0.293 | -0.089 |
| day.3.sofa - model | -0.1462 | 0.0022 | -0.240 | -0.053 |
| day.3.sofa – saps2 | 0.0447 | 0.3403 | -0.047 | 0.136 |

| ARDS-net | | | | |
| --- | --- | --- | --- | --- |
|  | Area Diff | P-value | lower.95 | upper.95 |
| saps2 - model | -0.1804 | 0.0000 | -0.238 | -0.123 |
| apache2 - model | -0.1955 | 0.0000 | -0.255 | -0.136 |
| apache2 - saps2 | -0.0150 | 0.3846 | -0.049 | 0.019 |

| KCLIP | | | | |
| --- | --- | --- | --- | --- |
|  | Area Diff | P-value | lower.95 | upper.95 |
| saps2 - model | -0.0162 | 0.6553 | -0.087 | 0.055 |

List of participants in the second international mechanical ventilation study

Argentina: **Coordinators**: Carlos Apezteguia (Hospital Prof. A. Posadas, El Palomar, Buenos Aires) and Pablo Desmery (Sanatorio Mitre, Buenos Aires).

A. Sarasino and D. Ceraso (Hospital Dr. Juan A. Fernández, Buenos Aires), D. Pezzola and F.Villarejo (Hospital Prof. A. Posadas, El Palomar), C. Cozzani and M. Torres Boden (Hospital Dr. C. Argerich, Buenos Aires), C. Santos and E. Capparelli (Hospital Eva Perón, San Martín), M. Tavella and C. Irrazábal (Hospital de Clínicas José de San Martín, Buenos Aires), L. Cardonnet and A. Diez (Hospital Provincial del Centenario, Rosario), A. Giannelli and L. Vargas (Policlínico de Neuquén), M. Bustamante (Hospital Héroes de Malvinas, Merlo), E. Turchetto (Hospital Privado de la Comunidad, Mar del Plata), J. Teves and O. Elefante (Hospital Oscar Alende, Mar del Plata), C. Sola and J. Mele (Hospital Dr. José Penna, Bahía Blanca), V. Sciuto and P. Grana (Hospital Provincial de Neuquén), G. Jannello and R. Valentini (CEMIC, Buenos Aires), S. Ilutovich (Sanatorio Mitre, Buenos Aires), L. Huespe Gardel (Hospital Escuela José F. de San Martín, Corrientes), J. Scapellato and E. Orsini (Hospital F. Santojanni, Buenos Aires), G. Agüero and Á. Sánchez (Policlínico Regional J. Perón, Mercedes), R. Fernández and L. Villalobos Castañeda (Hospital Italiano, Buenos Aires), F. González and E.Estenssoro (Hospital General San Martín, La Plata), S. Lasdica (Hospital Privado del Sur, Bahía Blanca), A. Gómez and J. Scapellato (Clínica de la Esperanza, Buenos Aires), P. Pratesi (Hospital Universitario Austral, Pilar), M. Blasco and F. Villarejo (Clínica Olivos, Olivos), G. Olarte and C. Bevilacqua (Clínica Modelo de Morón / Hospital San Juan de Dios, R.Mejía), M. Quinteros (Sanatorio San Lucas, San Isidro)

P. Ripoll (Clínica La Sagrada Familia, Buenos Aires), S. Filippus (Clínica del Valle, Comodoro Rivadavia), F. Guzman Díaz and M.Deheza (Hospital B. Rivadavia, Buenos Aires), E. García and J. Arrieta (Hospital Regional de Comodoro Rivadavia), P. Pardo and J. Neira (Sanatorio de la Trinidad de Palermo, Buenos Aires), J. Núñez and F. Pálizas (Clínica Bazterrica, Buenos Aires), A. Ciccolini and G.Murias (Sanatorio Santa Isabel, Buenos Aires), W. Vázquez and M. Grilli (Hospital Español de Mendoza, Godoy Cruz), F. Chertcoff and E. Soloaga (Hospital Británico, Buenos Aires), D. Vargas and J. Berón (Hospital Pablo Soria, San Salvador de Jujuy), A. Maceira and P. Schoon (Hospital Prof. Luis Güemes, Haedo), D. Pina (Sanatorio Franchín, Buenos Aires), E. Sobrino and A.Raimondi (Sanatorio Mater Dei, Buenos Aires), E. De Vito (IIM Alfredo Lanari, Buenos Aires).

Belgium: M. Malbrain (Ziekenhuis Netwerk, Antwerpen)

Bolivia: **Coordinator:** Freddy Sandi Lora (Hospital obrero N° 1, La Paz)

A. Lavandez and C. Alfaro (Complejo Hospitalario Viedma, La Paz), J. Guerra (Instituto gastroenterológico boliviano japonés, Santa Cruz).

Canada: **Coordinators** Niall D.Ferguson (Toronto Hospital Western Division) and Maureen O. Meade (McMaster University)

J.T. Granton (Toronto General Hospital), S. E. Lapinsky (Mount Sinai, Toronto), J. Meyer (St. Joseph´s Hospital, Toronto), D.C. Scales (St. Michael´s Hospital, Toronto), R.A. Fowler (Sunnybrook Health Sciences Centre, Toronto), B. Kashin (William Osler Health Centre, Brampton, Ontario), D. J. Cook (St. Joseph's Healthcare),

Colombia: **Coordinator** Marco A. González (Clínica Medellín y Universidad Pontificia Bolivariana, Medellín)

A. Guerra (Hospital General de Medellín and Clínica SOMA, Medellín), C. Cadavid (Hospital Pablo Tobón Uribe, Medellín), R. Panesso (Clínica Las Américas, Medellín), M.Granados (Clínica Valle del Lilli, Cali), C. Dueñas (Hospital Bocagrande, Cartagena), F. Molina (Clínica Bolivariana, Medellín), R. Camargo (Clínica General del Norte de Barranquilla), G. Ortiz (Hospital de Santa Clara, Bogotá), M. Gómez (Hospital de San José).

Chile: **Coordinator:** Vinko Tomicic (Clínica Alemana de Santiago)

L.Soto (Instituto Nacional del Tórax, Santiago), C. Romero (Hospital Clínico Pontificia Universidad Católica, Santiago), M.Teresa Caballero and L. Chiang (Hospital naval almirante NEF), E. Poch (Instituto de Neurocirugía), J. Canteros Gatica (Hospital Curico), H. Ugarte (Hospital de Coquimbo), M. Calvo, C. Vargas and M.Yacsich. (Hospital Regional de Valdivia), E. Tobar (Hospital Clínico de la Universidad de Chile, Santiago), J. G. Urra (Clínica Alemana de Temuco)

England: **Coordinator:** Peter Nightingale (Wythenshawe Hospital, Manchester)

J. Hunter (Macclesfield District General Hospital, Macclesfield), J. Hunter (Rotherdam District General Hospital, Rotherdam), S. Mousdale (Blackburn Royal Infimary, Blackburn), J. Harper (Royal Liverpool University Hospital, Liverpool), A. Conn (Wansbeck General Hospital, Ashington), D. Higgins (Southend Hospital, Westcliffe-on-Sea), D.Jayson **(**Southport & Formby District General Hospital, Southport), D. Hawkins (North Staffordshire Hospital, Stoke on Trent).

Ecuador: **Coordinator:** Manuel Jibaja (Hospital Militar de Quito)

G. Paredes and E. Bazantes (Hospital Enrique Garcés, Quito), P. Jiménez (Hospital Carlos Andrade Martín, Quito), J. Vergara and L. González (Hospital Luis Vernaza Valdez, Guayaquil)

France: **Coordinators** Laurent Brochard (Hôpital Henri Mondor, Créteil) and Arnaud Thille (Hôpital Henri Mondor, Créteil).

L. Mallet (Centre Hospitalier D’Auch), P. Andrivet (Centre Médico-Chirurgical de Bligny, Bris-sous-Forges), O. Peyrouset (Hôpital Ambroise Paré, Boulogne Billancourt), I. Mohammedi (Hôpital Edouard Herriot, Lyon), E. Guerot (Hôpital Européen Georges Pompidou , Paris), N. Deye (Hôpital Lariboisière, Paris), S. Monsel and F. Bouvet (Hôpital Pitié Salpétrière, Paris), M. Darmon (Hôpital Saint Louis, Paris), M. Fartoukh and A. Harb (Hôpital Tenon, Paris), N. Anguel (Hôpital de Bicêtre, Kremlin-Bicêtre).

Germany:  **Coordinator:** Konstantinos Raymondos (Medizinische Hochschule Hannover)

A. Nowak, T. Pahlitzsch and K. F. Rothe (Krankenhaus Dresden-Friedrichstadt), M. Ragaller and T. Koch (Universitaetsklinikum Carl Gustav Carus Dresden), G. Sterzel (Kreiskrankenhaus Loebau, Ebersbach), R. Wittich (Carl-Thiem-Klinikum Cottbus gGmbH),  K. Rudolph and J. Raumanns (St. Elisabeth gGmbH Leipzig), U. Grueneisen and F. Stupacher (Bundeswehrkrankenhaus Leipzig), H. Bromber, G. Leonhardt and J. Soukup (Universitaetsklinikum der Martin-Luther-Universitaet Halle-Wittenberg), C. Wuttke (Krankenhaus St. Elisabeth und St. Barbara Halle, Saale), M. Holler (Staedtisches Krankenhaus Martha-Maria Halle-Doelau gGmbH), J. Haberkorn (Georgius-Agricola-Klinikum Zeitz), P. Jehle   (Paul-Gerhard-Stiftung, Lutherstadt Wittenberg), B. Albrecht (Zeisigwaldkliniken Bethanien Chemnitz), Klut (Kreiskrankenhaus Rochlitz), H. J. Hartung (Vivantes Krankenhaus am Urban, Berlin-Kreuzberg), H. Gerlach (Vivantes-Klinikum Neukoelln, Berlin), T. Henneberg, S. Weber-Carstens, K. Haid, and C. Melzer-Gartzke, M. Oppert (Charité Universitaetsklinikum, Campus Virchow, Berlin), M. Reffenberg (Lungenklinik Heckeshorn, Berlin), Ch. Werel and  A. Kopietz (Klinikum Barnim GmbH, Werner Forßmann Krankenhaus, Eberswalde), T. Nippraschk and D. Hoffmeister (Ruppiner Klinikum GmbH, Neuruppin), M. Schneider (Dietrich-Bonhoeffer-Klinikum-Neubrandenburg), D. A. Vagts and G. Noeldge-Schomburg (Medizinische Fakultaet der Universitaet Rostock), G. Savinski and T. Kloess (Allgemeines Krankenhaus Harburg, Hamburg), C. Frenkel, D. Yakisan, H. Schroeder and C. Daniels (Staedtisches Klinikum Lueneburg), B. Sedemund-Adib (Universitaetsklinikum Schleswig Holstein - Campus Luebeck), S. Krueper (Klinikum Hannover Nordstadt),   J. Ahrens, U. Molitoris and  K. Johanning  (Medizinische Hochschule Hannover), D. Korth and  W. Seitz (Kreiskrankenhaus Hameln), J. Kleideiter and P. Palomino (Staedtische Kliniken Bielefeld gGmbH),   A. Lunkeit and Schlechtweg (Klinikum Bad Salzungen gGmbH),   M. Quintel (Universitaetsklinikum der Georg-August-Universitaet Goettingen), Schild and  C.P. Criée (Evangelisches Krankenhaus Goettingen-Weende e.V., Bovenden-Lenglern), M. Bund (Albert-Schweitzer-Krankenhaus Northeim), M. Hundt, U. Schulze and J. Kolle (Kreiskrankenhaus Charlottenstift, Stadtoldendorf), J. Offensand, S. Youssef, and J.P. Juvana (Klinikum Salzgitter GMBH), W. Seyde (Staedtisches Klinikum Wolfenbuettel),   T. Luecke and A. Gruener (Universitaetsklinikum Mannheim), E. Calzia (Universitaetsklinikum fur Anasthesiologie, Ulm), J. Heine, M. Borth, U. von Leitner and M. Hoffmann (Dr. Herbert-Nieper-Krankenhaus-Goslar), W. Brandt (Universitaetsklinikum Magdeburg), A. Keller and S. Scieszka (Krankenhaus Neuwerk, Moenchengladbach), E. Schroeder and F. L. Deres (Kreiskrankenhaus Dormagen), M. Burrichter, T. Bernhardt and W. Wilhelm (St.-Marien-Hospital, Luenen), M. Beiderlinden (Universitaetklinikum Essen), H. Steiniger and V. Weißkopf (Ruhrlandklinik, Essen), H. Militzer (Evangelisches und Johanniter Klinikum, Dinslaken), K. Eicker and F. Hinder (Universitaetsklinikum Muenster), C. Weilbach and M. Raab (St. Josefs-Stift Cloppenburg),   Ragaymutu (Kliniken der Stadt Koeln Krankenhaus Holweide),  T. Moellhoff and K. Tsompanidis (Katholische Stiftung Marienhospital Aachen), D. Henzler and R. Kuhlen (Universitaetsklinikum Aachen), H. Wrigge, C. Putensen and F. L. Dumoulin (Universitaetsklinikum Bonn), M. Foedisch and J. Busch (Evangelisches Waldkrankenhaus Bad Godesberg gGmbH, Bonn), W. Theelen (St. Johannes-Krankenhaus Troisdorf), A. Deller  (Krankenhaus der Barmherzigen Brueder, Trier), W. Baier (St. Nikolaus-Stiftshospital GmbH, Andernach), Eller (Staedt. Hellmig-Krankenhaus, Kamen), K. Schwarke (Evang. Krankenhaus Schwerte GmbH), Buettner (Evangelisches Krankenhaus Elisabethenstift gGmbH, Darmstadt), K. P. Wresch and K. Steidel (St.-Vincentius-Krankenhaus Speyer), J. F. Meyer (Universitaetsklinikum der Ruprecht-Karls-Universitaet Heidelberg), M. Layer (Thoraxklinik Heidelberg gGmbH), G. Meinhardt (Robert-Bosch-Krankenhaus, Stuttgart), J. Fritschi and P. Zaar (Ermstalklinik Staedtisches Krankenhaus Sindelfingen), H. P. Stegbauer (Kreiskrankenhaus Leonberg), Tumbass and S. Hahn (Ermstalklinik Bad Urach), H. Mende, M. Fischer, J. Martin and  A. Assmann (Klinik am Eichert Goeppingen), V. Schoeffel, K. van Deyk and S. Seyboth (Stadtklinik Baden-Baden), H. Kerger and Ernst (Evangelisches Diakoniekrankenhaus, Freiburg), H. F. Ginz (Kreiskrankenhaus Loerrach),   F. Brettner (Krankenhaus der Barmherzigen Brueder, Muenchen), O. Karg (ASKLEPIOS Fachkliniken Muenchen-Gauting), M. Glaser and T. P. Zucker (Klinikum Traunstein), J. Jahn and A. Schneider (Fachkliniken Wangen), M. Burkert (Bundeswehrkrankenhaus Ulm), H. Kuenzig and T. Bein (Klinikum der Universitaet Regensburg), A. Speicher (Krankenhaus der Barmherzigen Brueder, Regensburg), J. Brederlau, E. Kaufmann, F. Schuster and C. Soellmann (Universitaetsklinik Wuerzburg), S. Frenzel and L. Pfeiffer (Unstrut-Hainich Kreiskrankenhaus Muehlhausen), S. Weber-Carstens, K. Haid, C. Melzer-Gartzke, C. von Heymann and B. Temmesfeld (Charité Universitaetsklinikum, Campus Mitte, Berlin).

Greece: **Coordinator:** Dimitrios Matamis (Papageorgiou General Hospital, Thessaloniki).

H. Mouloudi (Ippokration General Hospital, Athens)

Italy: **Coordinator:** Paolo Pelosi (Ospedale di Circolo di Varese)

A.Pesenti and N. Rossi (Ospedale San Gerardo, Monza), D. Chiumello and L. Gattinoni (Ospedale Maggiore Policlinico, Milano), P. Severgnini (Ospedale di Circolo di Varese), R. Fumagalli and A. Nikiforov (Ospedali Riuniti di Bergamo), S.Grasso (Ospedale di Venere,  Bari).

Mexico: **Coordinator**: José Elizalde (Hospital ABC, México DF)

P. Cerda (Centro Médico de las Américas, Mérida), R. Mercado (Hospital Universitario de Monterrey), J.Albe Castañón (Instituto mexicano del seguro social HECMNS XXI, México DF).

Netherlands: Michael Kuiper, P.H.M. Egbers and M. Koopmans (Medical Center Leeuwarden)

Peru: **Coordinator:** Ana María Montañez

M. Contardo, J. Cerna and R.Roldán (Hospital Edgardo Rebagliati Martins, Jesús María), J.Zevallos and S.Alcabes (Hospital Guillermo Almenara Irigoyen, La Victoria), C.Salcedo and D.Bruzone (Hospital Nacional Daniel Alcides Carrión, Callao), J.Quiñones (Hospital de Emergencias Grau, Lima), M.Suárez Lazo (Hospital Nacional Hipólito Unanue, El Agustino), A.Cifuentes (Hospital de Emergencias José Casimiro Ulloa, Miraflores), M.Mayorga (Clínica San Pablo, Lima).

Portugal: **Coordinator:** Rui Moreno (Hospital de Santo António dos Capuchos, Lisboa)

P.Casanova (Hospitais da Universidade de Coimbra), R. Matos and A.L. Jardim (Hospital de Santo António dos Capuchos, UCIP, Lisboa), A. Godinho (Hospital dos SAMS, UCI, Lisboa), P. Póvoa (Hospital São Francisco Xavier, UCIM, Lisboa), P. Coutinho (Centro Hospitalar de Coimbra), L. Reis (Hospital de São José, Unidade de Urgência Médica, Lisboa).

Saudi Arabia: **Coordinator:** Yaseen Arabi (King Fahad National Guard Hospital)

N.Abouchala (King Faisal Hospital), F. Hameed (King Khalid National Guard Hospital)

Spain: **Coordinators:** Nicolas Nin and Eva Tejerina (Hospital Universitario de Getafe).

F. Gordo (Fundación Hospital de Alcorcón), R. Fernandez (Complejo Hospitalario Parc Taulí, Sabadell), R. de Pablo (Hospital Universitario Príncipe de Asturias, Alcalá de Henares), J. Ibañez (Hospital Son Dureta, Palma de Mallorca), E. Fernández Mondejar (Hospital Virgen de las Nieves, Granada), F. del Nogal (Hospital Severo Ochoa, Leganés), F. Taboada (Hospital Central de Asturias, Oviedo), A. García Jiménez (Hospital Arquitecto Marcide, El Ferrol), Ll. Cabré and J. Morillas (Hospital de Barcelona-SCIAS), S. Macias (Hospital General de Segovia), R. de Celis (Hospital de Galdakao), J. M.Añón (Hospital Virgen de la Luz, Cuenca),P. Ugarte (Hospital Marqués de Valdecilla, Santander), T. Mut (Hospital de la Plana, Vila-Real), J. Diarte (Complejo Hospitalario de Ciudad Real), V. Sagredo (Hospital Clínico de Salamanca), M. Valledor (Hospital San Agustín, Avilés), G.González and L. Rodríguez (Hospital Morales Meseguer, Murcia), V.Parra and E. Gómez (Hospital de Sagunto), F. Jara (Hospital Mutua de Terrassa), J.M. Quiroga (Hospital de Cabueñes, Gijón), L. Arnaiz (Hospital Clínico Universitario de San Carlos, Madrid), Á. Ayensa (Hospital Virgen de la Salud, Toledo), F. Suárez Sippman (Fundación Jiménez Díaz), F. Charizosa (Hospital General de Jerez de la Frontera), J. A. Rodríguez Sarría (Hospital de Elda), C. Homs (Hospital San Jorge, Huesca), A. Díaz Lamas (Hospital Cristal Piñor, Ourense), M. León (Hospital Arnau de Vilanova, Lleida), J. Allegue (Hospital Nuestra Señora del Rosell, Cartagena), M.Ruano (Hospital La Fe, Valencia).

Tunicia: **Coordinator:** Fekri Abroug **(**Fattouma Bourguiba Monastir)

M.Besbes, J. Ben Khelil, K. Belkhouja and K. BenRomdhane (Hospital Abderrahmane Mami, Ariana), S. Ben Lakhal, S. Abdellatif and K. Bousselmi (La Rabta Tunis), M.Amamou and H.Thabet (CAMUR), L. Besbes and N. Nciri (Fattouma Bourguiba Monastir), M.Bouaziz, H. Kallel and M. Bahloul (Habib Bourguiba Sfax), S. ElAtrous, S.Merghli and M. Feki Hassen (Tahar Sfar Mahdia).

Turkey: **Coordinator: Nahit Cakar** (Dokuz Eylun University, Istanbul).

R. Iscimen (Uludag University School of Medicine, Bursa), M. Kyzylkaya (College of medicine, Ataturk University, Erzurum), B. Yelken (Osmangazi University, Eskisemir), I. Kati (Medical Faculty of Yuzuncu Yil University, Van) , T. Guldem (Haydarpasa Numune Teaching and Research Hospital, Istambul), U. Koca (Dokuz Eylun University, Istanbul), M.Cicek (Inonu University of Medical Faculty, Malatya).

United States: **Coordinator:** Antonio Anzueto (University of Texas Health Science Center, San Antonio, Texas)

A.C. Arroliga (Cleveland Clinic, Cleveland), M Ali, O. Gajic, (Mayo Clinic, Rochester), Ch. Burger and L. Gambino D. (Mayo Clinic, Jacksonville), Ost, A.Fein, A. Kyprianou, L. Shulman and S. Chang (North Shore University Hospital, New York), J.S. Steingrub, M.A. Tidswell and K. Kozikowski (Baystate Medical Center, Springfield), C.A. Piquette and L.Morrow (Creighton University Medical Center, Nebraska), P.Scheinberg and J.Green (Saint Joseph’s Hospital, Atlanta), L.Penogreen and K. Kannady (Georgia State University Kennestone), M. Moss, M. Mealer, and R.D. Restrepo (Grady Hospital Georgia, Atlanta), H.E. Fessler, R. Brower, D. Hager and A. Scully (John Hopkins University Hospital, Baltimore), J. Beamis, D.E. Craven andW. Miner (Lahey Clinic Medical Center, Burlington), S. Blosser, K. Miller, L.Cornman and J.Breidinger (Penn State Hershey Medical Center, Hershey), J.T. Huggins and Ch.Strange (Medical University of South Carolina, Charleston), N.S. Hill and L.Lawler (Tufts-New England Medical Center, Boston), M. Rembert (Newark Beth Israel Medical Center), H.K. Donnelly, J.D. D’Amico, R.G. Wunderink, N.Queseda and J. Topin (Northwestern Memorial Home Health University, Chicago), G.T. Kinasewitz and G.L. Lee (University of Oklahoma Health Sciences Center, Oklahoma City), J.Walls and V. Zimmer (Presbyterian Healthcare, Charlotte), A.X. Freire (Regional Medical Center, Memphis), C. Steven and L. Caskey (Louisiana State University Health Sciences Center, Shreveport), R. Dhand and L.A. Despins (University Hospital and Clinics MU Healthcare, Columbia), R.Hyzy, R.E. Dechert,Carl Haas, D. Fickle (University of Michigan Medical Center), D. Marks and S. Benslimane (University of Texas Health Science Center, San Antonio), V.J. Cardenas Jr. (University of Texas Medical Branch Galveston), M.J. Wing and P.Krumpe (VA Sierra Nevada Health Care System, Reno), J. Truwit and M. Marshall (University of Virginia Health System, Charlottesville), D.L. Herr (Washington Hospital Center, Washington DC), RD Hite (Wake Forest Baptist Hospital Medical Center, Winston Salem), PJ McShane and KN Olivier (Wilford Hall Medical Center, Texas), KW Presberg (Froedtert & Medical College, Milwaukee).

Uruguay: **Coordinator:** Javier Hurtado (Cudam Sanatorio Colón, Sanatorio IMPASA and Hospital de Clínicas, Montevideo)

M. Borde, E.Echavarría, S. Gómez and M. Berón (Hospital Maciel, Montevideo), F. Villalba (Sanatorio Casa de Galicia, Montevideo), I. Porras (Sanatorio CASMU 2, Montevideo), P. Cardinal, C. Surraco and V. Navarrete (Sanatorio CASMU 4, Montevideo), F. Rodríguez and J.C. Bagattini (Hospital Británico, Montevideo), R. Garrido (Hospital Evangélico and Sanatorio IMPASA, Montevideo), S. Infanzón and J. Caraballo (Hospital Militar and CTI-SMI, Montevideo), C. Santos and A. García (Hospital de Clínicas, Montevideo), R. Cal (CTI-SMI, Montevideo), G. Pittini and J. Cabrera (Centro Nacional de Quemados, Montevideo), F. Bazzano and F. Domínguez (Hospital Pasteur, Colonia), P. Alzugaray, D. González and M.Machado (Sanatorio CAMOC, Carmelo), F.Torres (Sanatorio Mautone and Asistencial Medica de Maldonado, Maldonado), S. Mareque, M. Korintan, F.Mora, E. Altieri, E. Gianoni, C. Fregosi, A. Crossi, G. Larrarte (Sanatorio CAAMS, Soriano), O. Pereira (Sanatorio COMTA, Tacuarembó), J. Baraibar (Hospital Regional de Tacuarembó), A. Soler (Sanatorio COMEPA, Paysandú), M. Rodríguez Verde (Hospital Paysandú), M. Díaz (Hospital de Salto and Sanatorio Uruguay, Salto), J. Martínez Ramos (Sanatorio Uruguay, Salto), I. Iturralde, W. González and E. Cubas (Sanatorio CAMDEL, Minas), A. Cataldo (Sanatorio CAMEDUR, Durazno), O. Rocha (Sanatorio GREMEDA, Artigas), A.Deicas (Sanatorio CASMU 2 and Sanatorio CASMU 4)

Venezuela: **Coordinator** Gabriel D’Empaire (Hospital de Clínicas, Caracas).

R. Zerpa (Hospital Militar de Caracas), M. Narvez (Hospital Domingo Luciani, Caracas), F.Pérez (Hospital de Clínicas, Caracas), J. España (Hospital Universitario de Caracas).

**Participants in the National Heart, Lung, and Blood Institute (NHLBI) ARDS Clinical Trials Network were as follows**: *Investigators (principal investigators are marked with an asterisk): Cleveland Clinic Foundation* — H.P. Wiedemann,* A.C. Arroliga, C.J. Fisher, Jr., J.J. Komara, Jr., P. Periz-Trepichio; *Denver Health Medical Center* — P.E. Parsons; *Denver Veterans Affairs Medical Center* — C. Welsh; *Duke University Medical Center*— W.J. Fulkerson, Jr.,* N. MacIntyre, L. Mallatratt, M. Sebastian, J. Davies, E. Van Dyne, J. Govert; *Johns Hopkins Bayview Medical Center* — J. Sevransky, S. Murray; *Johns Hopkins Hospital* — R.G. Brower, D. Thompson, H.E. Fessler, S. Murray; *LDS Hospital* — A.H. Morris,* T. Clemmer, R. Davis, J. Orme, Jr., L. Weaver, C. Grissom, F. Thomas, M. Gleich (deceased); *McKay-Dee Hospital* — C. Lawton, J. D'Hulst;*MetroHealth Medical Center of Cleveland* — J.R. Peerless, C. Smith; *San Francisco General Hospital Medical Center* — R. Kallet, J.M. Luce; *Thomas Jefferson University Hospital* — J. Gottlieb, P. Park, A. Girod, L. Yannarell; *University of California, San Francisco* — M.A. Matthay,* M.D. Eisner, J. Luce, B. Daniel, T.J. Nuckton; *University of Colorado Health Sciences Center* — E. Abraham,* F. Piedalue, R. Jagusch, P. Miller, R. McIntyre, K.E. Greene; *University of Maryland*— H.J. Silverman,* C. Shanholtz, W. Corral; *University of Michigan* — G.B. Toews,* D. Arnoldi, R.H. Bartlett, R. Dechert, C. Watts; *University of Pennsylvania* — P.N. Lanken,* J.D. Christie, B. Finkel, B.D. Fuchs, C.W. Hanson, III, P.M. Reilly, M.B. Shapiro; *University of Utah Hospital* — R. Barton, M. Mone; *University of Washington/Harborview Medical Center* — L.D. Hudson,* G. Carter, C.L. Cooper, A. Hiemstra, R.V. Maier, K.P. Steinberg, Margaret Neff, Patricia Berry-Bell; *Utah Valley Regional Medical Center* — T. Hill, P. Thaut; *Vanderbilt University* — A.P. Wheeler,* G. Bernard,*B. Christman, S. Bozeman, T. Swope, L.B. Ware; *Clinical Coordinating Center, Massachusetts General Hospital, Harvard Medical School*— D.A. Schoenfeld,* B.T. Thompson, M. Ancukiewicz, D. Hayden, MA, F. Molay, N. Ringwood, C. Oldmixon, A. Korpak, R. Morse; *NHLBI Staff* — D.B. Gail, A. Harabin,* P. Lew, M. Waclawiw*; *Steering Committee* — G.R. Bernard (chair);*Data and Safety Monitoring Board* — R.G. Spragg (chair), J. Boyett, J. Kelley, K. Leeper, M. Gray Secundy, A.S. Slutsky, B. Turnbull; *Protocol Review Committee* — J.G.N. Garcia (chair), S.S. Emerson, S.K. Pingleton, M.D. Shasby, W.J. Sibbald
